# Teaching anxiety among novice Chinese language teachers: manifestations, causes, and interventions—a mixed-methods study in Guangdong Province, China

**DOI:** 10.3389/fpsyg.2026.1796834

**Published:** 2026-03-25

**Authors:** Kangshu Xiao

**Affiliations:** School of Literature and Communication, Zhaoqing University, Zhaoqing, China

**Keywords:** intervention experiment, novice Chinese language teachers, situational anxiety outbursts, support model, teaching anxiety

## Abstract

This mixed-methods study investigates the manifestations, underlying causes, and potential interventions for teaching anxiety among novice Chinese language teachers. Drawing on data from 1,248 questionnaires and 260 open-ended responses collected in Guangdong Province, China, from 2024 to 2025, the research examines the dimensions, triggers, and dynamic progression of such anxiety. Key findings reveal that (1) while overall state anxiety was manageable, intense situational outbursts frequently occurred during public demonstrations, leadership observations, and when managing students with special needs; (2) among the five identified anxiety dimensions, evaluation and assessment anxiety (89.9%) and teaching content anxiety (82.0%) were most prevalent; (3) the relationship between teaching experience and anxiety was nonlinear, characterized by a sharp increase peaking at four months (STAI-State = 5.1), followed by a decline and a secondary rise after 18 months associated with professional development pressures; and (4) teachers in rural schools, those serving as homeroom advisors, and those with high weekly teaching loads faced significantly elevated anxiety risk. An intervention experiment testing a support model based on contextual buffering, instructional load reduction, technological integration, and institutional safeguards demonstrated significant efficacy, reducing the average anxiety score among participating teachers from 5.1 to 2.4 and decreasing turnover intention by 28%. This study provides robust, evidence-based support for developing targeted, data-driven support systems for novice language teachers.

## 1 Introduction

Amidst the accelerating modernization and digital transformation of education, language teachers in China face unprecedented professional challenges. The 2022 “Strong Teacher Plan for Basic Education in the New Era, “which was jointly issued by eight ministries, including the Ministry of Education, explicitly calls for Chinese language teachers to increase their disciplinary teaching proficiency and interdisciplinary integration skills and advocates for approaches such as “parallel lesson study” and “case study” to resolve practical teaching issues (Ministry of Education, Central Propaganda Department, Central Organization Department, National Development and Reform Commission, Ministry of Finance, Ministry of Human Resources and Social Security, Ministry of Housing and Urban-Rural Development, & National Rural Revitalization Administration, 2022). Compounding these demands, national projections indicate that 72% of newly hired language teachers will enter the profession by approximately 2025. These novice teachers, who are undergoing a triple transition-adapting to their subject, grade level, and professional role, are experiencing significant anxiety.

Research suggests that teaching anxiety among novice Chinese language teachers—specifically those teaching Chinese as a mother tongue in K-12 schools— has unique disciplinary characteristics. The 2022 Compulsory Education Chinese Curriculum Standards introduce advanced requirements such as “whole-book reading,” “cross-media writing,” and “task-based unit design” (Ministry of Education of the People’s Republic of China, 2022). These expectations, coupled with pressures from digital transformation, render the Chinese language classroom a domain of intense emotional labor (Yin et al., 2019). Compared with other subjects, Chinese language teaching involves a triple cognitive load stemming from textual polysemy, subjective assessment, and rapid technological iteration (Sweller et al., 2019). This leads to a predominant dilemma for novice teachers, torn between overinterpretation and teaching narrowly to the test in areas such as classical text analysis and writing instruction (Tsui, 2009). This disciplinary specificity results in a significantly higher incidence of anxiety events among novice Chinese teachers than among their peers in other subjects, with anxiety more readily transmitted through classroom interactions, forming a negative feedback loop (Zembylas, 2003).

A significant methodological gap persists in the current literature. Existing studies predominantly rely on single-method approaches, utilizing either standardized scales or qualitative case descriptions, and lack mixed-methods designs that examine the interaction between subject area, grade level, and teaching experience. Domestic research often focuses on a one-dimensional perspective of psychological symptoms or institutional critique. While international studies have incorporated physiological indicators and longitudinal tracking, they frequently overlook disciplinary differences and lack adaptation to the local context. The data reveal that 62% of novice teachers perceive a complete disconnect between their university training and classroom practice, revealing a 24-month knowledge gap between teacher education and frontline teaching. This theory-practice divide renders existing intervention measures fragmented and ineffective.

This context of policy and practical demands creates an imperative for innovative research. The “Strong Teacher Plan” specifically emphasizes the establishment of a “support system for novice teachers.” The convergence of the predicted 2025 teacher shortfall and the ongoing process of digital transformation make the construction of a data-driven, precise support model an urgent priority.

This study departs from unidimensional approaches to anxiety research by making theoretical contributions in three key areas. First, this work presents a tripartite “identity-emotion-institution” explanatory framework, revealing the mechanisms of pressure stemming from the collapse of the idealized teacher image, the alienation of emotional labor, and institutional disciplining. Second, it develops a “volcano model” of anxiety structure, comprising “situational eruption, teaching experience residuals, and individual heterogeneity,” to precisely identify high-risk situations such as public demonstrations and leadership observations. Third, it designs a coordinated intervention path of “cognitive calibration, social adaptation, and institutional restructuring.” By utilizing a mixed-methods approach with 1,248 questionnaires, this study validates the effectiveness of a support model based on contextual buffering, instructional load reduction, technological integration, and institutional safeguards.

In total, 1,248 novice Chinese language teachers were sampled from 27 districts and counties in Guangdong Province, covering diverse urban and rural school types. By employing the STAI State Anxiety Inventory, microbehavioral classroom coding, and a three-level NVivo coding process, a triangulated verification system integrating “data, narratives, and behavior” was established. By employing a mixed-methods approach with 1,248 questionnaires, this study validates the effectiveness of a support model based on contextual buffering, instructional load reduction, technological integration, and institutional safeguards. The risk of high anxiety was 24, 63, and 41% greater for rural teachers, homeroom advisors, and those with high teaching loads, respectively. These findings identify precise targets for mitigating high-intensity situational anxiety and establish an empirical foundation for building a lifelong teacher development support system.

By innovatively integrating teacher education research with education policy analysis, this study builds a theoretical model of the interaction between disciplinary specificities and the institutional environment while developing a replicable support system for stress reduction at the practical level. The research outcomes not only respond to the top-level design requirements of the “Strong Teacher Plan” for novice teacher cultivation but also provide an innovative pathway for optimizing classroom ecology under the “double reduction” policy. By elucidating the mechanisms generating and alleviating anxiety among novice Chinese language teachers, this study serves as a pivotal academic contribution to a paradigm shift in teacher education.

Research on teacher anxiety has evolved considerably since Kyriacou and Sutcliffe, (1977) seminal work provided an operational definition (Kyriacou and Sutcliffe, 1977). The field has progressed from single-factor models to multidimensional frameworks encompassing evaluation, discipline, technology, and emotional labor (Yurtseven and Sara, 2024). Internationally, studies often combine large-scale surveys with physiological measures, consistently demonstrating a nonlinear decline in anxiety with teaching experience but largely overlooking discipline-specific variations.

In contrast, research within the Chinese context has focused more on localized stressors. Recent domestic studies concentrating on novice teachers identify administrative tasks, unannounced classroom observations (“drop-in visits”), and high-stakes academic evaluation as primary pressure sources (Du, 2023). However, these studies are frequently limited by small sample sizes (*n* < 300), generic measurement tools, and a lack of longitudinal causal evidence.

Within this landscape, investigating disciplinary differences is crucial. As a mother-tongue discipline characterized by high textual uncertainty, Chinese language teaching presents a unique “pluralistic interpretation versus standard answer” tension, imposing a distinct emotional load on novices. Compared with routine teaching, the culture of public demonstration lessons transforms classrooms into “competitive arenas,” elevating state anxiety scores by 1.2–1.8 points (Li, 2024). Furthermore, compared with their urban counterparts, the digital transformation that introduces cross-media writing and smart classroom operations has resulted in rural teachers reporting 24 percentage points higher technological anxiety (Zhao and Dai, 2024). Despite their importance, these issues are often addressed sporadically in policy commentaries, lacking systematic empirical investigation.

Research on emotional labor further illuminates this context. Confucian norms of “teacher dignity” amplify the demand for emotional suppression, with surface acting showing a significant positive correlation with anxiety (Gao and Cai, 2025). While organizational support, such as mentorship programs and emotional leave, is theorized as a potential buffer, its pathways for alleviating anxiety remain largely hypothetical and lack rigorous validation through randomized controlled trials.

While the aforementioned studies provide valuable insights into the localized stressors within the Chinese context, it is imperative to situate this research within the broader international discourse on teacher development and well-being. Internationally, research on teacher anxiety has evolved from a focus on unidimensional stress models to multifaceted frameworks that acknowledge the dynamic interplay between personal resources and contextual demands. The job demands-resources (JD-R) model (Bakker and Demerouti, 2007) offers a particularly relevant heuristic framework; it posits that burnout and negative psychological outcomes arise from high job demands (e.g., workload and emotional demands), which are buffered by job resources (e.g., social support and autonomy). Previous studies in Western contexts have applied the JD-R model to teaching, identifying demands such as student misbehavior and bureaucratic tasks and resources such as collegial support and supervisory coaching (Hakanen et al., 2006). However, the specific configuration of demands and resources for novice language teachers in a high-stakes, rapidly digitalizing education system such as that in China remains underexplored.

Furthermore, seminal stage models of teacher development, such as that proposed by Fuller and Bown (Fuller and Bown, 1975; Huberman, 1989), suggest a linear progression of teacher concerns from self (survival, adequacy) to task (instructional management) and, finally, to impact (student learning). While this model provides a foundational understanding of the novice phase, recent international longitudinal studies suggest a more complex, nonlinear emotional trajectory. For instance, the work of Yurtseven and Sara (2024) on teacher emotions identifies a potential “second peak” of anxiety related to professional development pressures after the initial survival phase, a phenomenon not fully captured by traditional linear models. This study seeks to examine whether such a nonlinear curve, characterized by residual and resurgent anxieties, manifests among novice Chinese language teachers, thereby testing and potentially refining these established models within a distinct cultural and disciplinary context.

Several studies have specifically examined anxiety among language teachers. For instance, Horwitz (1996) highlighted the emotional challenges faced by foreign language teachers, while Mercer and Kostoulas (2018) explored the emotional labor involved in language teaching. In the context of Chinese as a mother tongue, recent work by Zhang (2020) and Li (2024) has begun to explore the unique pressures of teaching classical Chinese and managing public demonstrations. However, these studies are often limited in scope and lack large-scale empirical validation. The present study builds on this emerging body of research by offering a comprehensive, mixed-methods analysis of anxiety among novice L1 Chinese language teachers.

In summary, the literature reveals four primary gaps: disciplinary absence, methodological singularity, mechanistic ambiguity, and fragmented evidence. First, it fails to integrate the “textual openness of Chinese, subjectivity of evaluation, and disruptive nature of technology” into a cohesive analytical framework for anxiety. Second, large-scale, mixed-methods studies that form a closed loop of “quantitative profiling, behavioral verification, and intervention experimentation” for anxiety-inducing situations are scarce. Third, longitudinal causal evidence is lacking for both the “anxiety-teaching experience curve” and the impact of “systemic interventions on anxiety changes.”

Recently, international scholars have begun to acknowledge the phenomenon of shifting emotional challenges during the early career stages. Longitudinal research by Klusmann and colleagues (e.g., Klusmann et al., 2008; Klusmann et al., 2016) suggests that teacher emotions often evolve from initial concerns regarding classroom management and ‘survival’ to more complex anxieties related to professional effectiveness and pedagogical depth as teachers gain experience. While the timing of this transition varies, studies indicate a critical period within the first two years of entry where support needs change significantly. These findings resonate with the ‘second peak’ observed in our LOWESS curve, strongly suggesting that support systems must dynamically adapt to different career stages. (Richter et al., 2011; Richter et al., 2013; Klusmann et al., 2008).

## 2 Materials and methods

In this study, novice teachers were defined as those with no more than one year of teaching experience currently assigned to Chinese language instruction. This study involved human participants and was conducted in accordance with the ethical standards of the institutional and national research committee and with the 1964 Helsinki declaration and its later amendments. The research protocol received formal approval from the ZhaoQing University Institutional Review Board (IRB) prior to data collection. All the participants were provided with detailed information about the study’s purpose, procedures, and rights. Written informed consent was obtained from each teacher before they participated in the survey, ensuring that their involvement was entirely voluntary. All the data were anonymized to protect participant confidentiality. The survey included open-ended questions that invited participants to provide narrative accounts of their teaching anxiety experiences.

The survey was designed to follow a “low-disguise, high-context” principle to enhance the authenticity and validity of the data. “Low-disguise” signifies that the research purpose—investigating teaching anxiety—was transparently communicated to participants, reducing evaluation apprehension and fostering trust, which discourages the provision of socially desirable but inaccurate responses. “High-context” refers to the framing of questions within rich, concrete situational vignettes (e.g., “Recall your most recent public demonstration lesson”) rather than asking about abstract or general feelings. This approach leverages episodic memory, making it easier for teachers to access and report their genuine emotional and physiological experiences, thereby mitigating recall bias and yielding more behaviorally grounded and contextually nuanced data.

A stratified sampling approach was employed, ensuring proportional representation from urban, town, and rural schools (approximately one-third each), coupled with individual saturation sampling.

The survey included the State–Trait Anxiety Inventory (STAI-State), the Maslach Burnout Inventory-Educators Survey (MBI-ES), the Teachers’ Sense of Efficacy Scale (TSES), and a bespoke Content Anxiety Scale developed for this study (see Appendix for the full questionnaire). The Content Anxiety Scale comprised five items rated on a 5-point Likert scale and showed good internal consistency (Cronbach’s *α* = 0.84).

Quantitative analysis utilized the STAI-State Anxiety Scale, exploratory factor analysis, and locally weighted scatterplot smoothing (LOWESS). Qualitative data underwent a three-level coding process (open, axial, and selective) using NVivo.

### 2.1 Intervention experiment: design and implementation

To empirically validate the proposed support framework, a quasiexperimental intervention was designed and implemented in 20 volunteer schools (10 experimental, 10 control) from the sample pool. The intervention, underpinned by the four-component model (contextual buffering, instructional load reduction, technological integration, and institutional safeguards), was operationalized through several concrete measures. The operational definitions, implementation processes, and measurement tools for key interventions are detailed below.

#### 2.1.1 AI feedback system for public lesson simulation

Operational Definition: This intervention involved a simulated teaching studio equipped with a 4 K recording system and an AI analytics platform. The AI system was programmed to code 22 discrete real-time behavioral indicators, which were categorized as follows:

Verbal Fluency: Frequency of filler words (e.g., “um,” “ah”), speech rate (syllables per minute), and pauses exceeding 2 s.

Nonverbal Cues: Gaze direction (toward students vs. screen/slide vs. evaluators), facial expressiveness (using a validated emotion recognition algorithm), and posture shifts.

Teaching Moves: Wait time after posing questions, ratio of open-ended to closed-ended questions.

Implementation process: Teachers in the experimental group conducted a mock public lesson in the studio. Immediately afterward, they received a personalized “Anxiety Heatmap Report” generated by the AI, which visually superimposed their anxiety-linked behaviors onto the lesson timeline. This was followed by a debriefing session with a trained mentor to set behavioral goals for improvement.

Measurement Tools: The efficacy of this intervention was measured by comparing the frequency of target behaviors (e.g., filler words, long pauses) in pre- and postintervention simulation videos. The STAI-State scale was administered immediately after the simulation to measure subjective anxiety.

#### 2.1.2 Emotional leave policy

Operational Definition: “Emotional leave” was defined as two days of paid, nonpunitive leave per month that teachers could use for self-directed psychological restoration or professional development activities without requiring medical reasons. Permissible activities included attending counseling sessions, engaging in peer observation for learning (not evaluation), and participating in well-being workshops or personal rest.

Implementation process: Teachers applied for leave through a simplified, confidential online system managed by the school’s HR department. The application required only the selected dates and a tick box for the general category of activity (e.g., “professional development,” “personal well-being”), protecting teacher privacy. School leadership committed to having no negative consequences for its usage.

Measurement Tools: The impact of emotional leave was primarily assessed using the Maslach Burnout Inventory-Educators Survey (MBI-ES), administered quarterly. Turnover intention was measured by a 3-item scale (e.g., “I often think about leaving the teaching profession”) on a 5-point Likert scale, with established reliability (Cronbach’s *α* = 0.89 in this sample).

#### 2.1.3 Co-teaching model for inclusive classrooms

Operational Definition: This intervention provided classrooms identified as having >15% of students with learning or behavioral difficulties with an additional resource teacher. The role of the resource teacher was defined as providing instructional support, managing minor disruptions, and implementing individualized behavior plans, allowing the main teacher to focus on core instruction.

Implementation process: Resource teachers were assigned a ratio of 0.3 full-time equivalents per eligible class. They participated in weekly planning meetings with the main teacher to align support strategies. In remote rural schools where a physical resource teacher was not feasible, an AI-based early warning service was piloted, which used classroom audio analysis to flag recurring patterns of disruption and alert the teacher via a discreet earpiece.

Measurement Tools: Effectiveness was measured by direct classroom observation coding the number and duration of instructional interruptions due to student behavior. Teacher self-efficacy for inclusive instruction was measured using a subscale of the Teachers’ Sense of Efficacy Scale (TSES).

#### 2.1.4 Prism interpretation toolkit

Operational Definition: A digital resource kit designed to reduce “teaching content anxiety.” For each core text in the curriculum, the toolkit provided three layers of annotation: Historical & Cultural Context (scholarly annotations), Curriculum Standards Alignment (mapping key text passages to examinable points), and Pedagogical Cases (multiple teaching examples demonstrating different interpretive approaches).

Implementation process: Teachers in the experimental group were given access to the toolkit and received one 3-h training session on its use. They were encouraged to integrate it into their regular lesson planning.

Measurement Tools: A bespoke “Content Anxiety Scale” was developed, comprising 5 items (e.g., “I feel confident in interpreting the deeper meaning of classical texts”) rated on a 5-point scale. This scale showed good internal consistency in the pilot test (*α* = 0.84). Usage data (frequency of access to the toolkit) were also logged.

### 2.2 Open-ended questions as qualitative data source

To complement the quantitative survey data, the questionnaire included six open-ended questions (see Appendix, Part Five, Questions 59–64). These questions invited teachers to describe memorable anxiety incidents, list anxiety-inducing scenarios, and suggest supportive measures. A total of 260 valid open-ended responses were collected, with an average length of 120 words per response. These narrative data were transcribed and analyzed using thematic analysis in NVivo, following a three-level coding process (open, axial, and selective). The qualitative findings derived from these responses were triangulated with survey data to enhance the validity and depth of the analysis.

In summary, the anxiety structure of novice Chinese teachers can be characterized as “generally controllable yet situationally explosive, multidimensional, and individually heterogeneous” and can be applied to a volcano. Public lessons, leadership observations, and managing special needs students constitute the three primary triggers. These findings provide precise targets for the subsequent support model based on contextual buffering, instructional load reduction, technological integration, and institutional safeguards.

## 3 Results and discussion

Teaching anxiety is not a straightforward product of individual psychological vulnerability but rather a “multilayered fault” reinforced by the interplay of cognitive gaps, cultural discipline, and institutional voids. This study analyzes the mechanisms that generate anxiety among novice Chinese language teachers across four interrelated dimensions: cognitive, sociocultural, institutional, and disciplinary.

### 3.1 Participant demographics and sample characteristics

From September 2024 to September 2025, the research team administered “low-disguise, high-context” anonymous surveys, yielding 1,248 valid questionnaires (63.3% from primary, 29.1% from junior secondary, and 7.6% from senior secondary schools; 72.6% female; 64.92% homeroom advisors; 38.7% rural). This was complemented by 260 in-depth open-ended responses, forming a robust mixed data source.

It should be noted that the sample distribution across school levels—with 63.3% from primary, 29.1% from junior secondary, and 7.6% from senior secondary schools—is not evenly balanced. This overrepresentation of primary school teachers reflects the actual employment distribution of novice Chinese language teachers in Guangdong Province, where hiring at the primary level substantially exceeds that at the secondary level due to demographic trends and education policy priorities. While this weighting enhances the ecological validity of our findings for the primary school context, it may limit the generalizability of conclusions to senior secondary settings, where curricular demands, textbook complexity, and student developmental stages differ considerably. Future research should intentionally oversample secondary teachers to enable cross-stage comparisons and a more comprehensive understanding of teaching anxiety across educational levels.

### 3.2 Overall profile: situational anxiety outbursts amid generally, low baselines

The overall mean STAI-State score for novice Chinese teachers was 1.61 (on a 10-point scale), suggesting a manageable baseline. However, anxiety exhibited a pronounced “situational-dependent” pattern. High-intensity anxiety was triggered primarily by specific scenarios: public demonstration lessons (89.92%), leadership observations (87.1%), and unannounced “drop-in” lessons (38.5%). In contrast, only 8.1% reported significant anxiety during routine teaching. Physiological markers substantiated these reports, with 25.8% experiencing insomnia, 19% reporting sweaty palms, and 14.1% reporting a loss of appetite, collectively delineating a “low-baseline, high-outburst” volcanic curve of anxiety.

### 3.3 Five-dimensional anxiety profile

Moving beyond classic tripartite models (Yang, 2021), which simplified anxiety into “lesson preparation, in-class teaching, and teacher-student relationships,” this study, based on 1,248 surveys, delineates in-class teaching anxiety into five distinct dimensions.

#### 3.3.1 Evaluation and assessment anxiety

Echoing macrolevel observations about time fragmentation in what sociologist Hartmut Rosa terms the ‘acceleration era’—a period characterized by technological acceleration, social change, and a heightened pace of life that intensifies time pressure and contributes to subjective time scarcity (Wang, 2024), our microlevel data reveal that novice teachers spend an average of 12.7 h preparing a single public lesson. When institutional time is consumed by administrative tasks, teachers compensate for using personal time after work and on weekends, directly transforming time pressure into preparation anxiety. Consequently, 90% overprepared for public lessons (averaging 12.7 h/lesson), with 87% revising their lesson plans five or more times. This manifests in classroom language characterized by a “recitation tone” and “mechanical interaction,” with postlesson reflections frequently containing phrases such as “fear of making mistakes” and “being criticized.”

#### 3.3.2 Student-related anxiety

The survey data revealed that student factors were the “most troubling” source of anxiety for 54.8% of the respondents. In 65.32% of the classes taught by participants, more than 15% of the students were identified as having learning difficulties or behavioral issues. For instance, students with ADHD were observed leaving their seats three or more times per lesson, causing teachers to interrupt instruction for an average of 5.8 min. Some teachers reported spending up to 49% of class time on behavior management, reducing curriculum coverage to just 52%.

#### 3.3.3 Classroom management anxiety

Classroom management was ranked as the “primary source of anxiety” by 28.2% of novice teachers. Caught between maintaining authority and showing care, 28% relied heavily on punitive point-deduction systems. Deteriorating teacher-student relationships were correlated with a sharp increase in STAI scores, from 3.2 to 6.8, over six months. A significant majority (72.58%) explicitly called for a reduced burden on new teachers.

#### 3.3.4 Teaching content anxiety

Consistent with Zhang Longrong’s findings (Zhang, 2020), novice teachers reported low confidence in grasping key and difficult parts of the textbook and significant anxiety regarding preparation time and volume. This study revealed specific manifestations: 82% felt anxious about teaching classical Chinese, 74% about writing instruction, and 68% about modern prose. Critically, 89% lacked confidence in interpreting the deeper meaning of texts.

#### 3.3.5 Technological anxiety

The national push for educational digitalization introduces significant technological demands (Franco-Buriticá et al., 2023). Consequently, 35.08% of novices reported anxiety about operating smart classroom systems, with rural teachers’ technological anxiety being 24 percentage points higher than that of their urban counterparts. Notably, all reported instances of equipment failure (e.g., black screens) during public lessons occurred in high-stakes evaluation contexts, creating a “dual assault” of technological and evaluation anxiety.

### 3.4 Teaching experience–anxiety curve

LOWESS regression revealed a nonlinear relationship between teaching experience and anxiety. Anxiety increased sharply during the first 0–3 months but peaked in the fourth month (STAI-State = 5.1). It then decreased and stabilized at approximately 12 months (STAI-State = 2.4). However, a “second peak” emerged after 18 months, attributed to rising “professional development anxiety,” indicating a necessary shift in support focus from initial “survival” to sustained “growth.”

### 3.5 High-risk teacher profiles

A multiple logistic regression model (high anxiety = 1) revealed several significant risk factors: serving as a homeroom advisor (OR = 2.31), having a weekly teaching load of ≥13 lessons (OR = 1.97), being a rural teacher (OR = 1.79), entering teaching without formal pedagogical training (OR = 1.56), and teaching in a school with a higher performance ranking (OR increased by 1.48 per rank). The model explained 42% of the variance (R^2^ = 0.42). Gendered differences in impression management were also observed: the percentage of time spent on appearance management increased from 15 to 52 min for female teachers, whereas the percentage of male teachers who wore dark suits and ties increased from 8 to 63% in high-stakes situations.

### 3.6 Micro-behavioral evidence

Analysis of 42 classroom videos revealed behavioral correlates of anxiety. During public lessons, pauses exceeding two seconds occurred 18 times per lesson on average-3.2 times more frequently than during routine lessons did. The use of filler words increased from 5.3 to 27 instances per lesson. Furthermore, upon the entrance of school leaders, teachers’ gaze toward the PPT screen increased from 52 to 78%, indicating a strategy of “technology-dependent emotional avoidance.”

### 3.7 Cognitive layer: the rupture between ideal and real self

Perfectionistic Self-Construction: Teacher education often prioritizes theoretical knowledge over situational application, leading 57% of novices to equate being a “good teacher” with being a “flawless, zero-error” professional. When unforeseen situations arise in the classroom, it triggers a sense of loss of control-epitomized by the experience of “having prepared for five hours yet still omitting a third of the content.” The first public demonstration lesson often becomes a trigger for self-doubt.

Negative Efficacy Cycle: There is a pronounced lack of Bandura-defined “mastery experiences.” Juggling the roles of homeroom advisors, high weekly teaching loads (≥13 periods), and administrative tasks leaves 64% of teachers without a sense of fully completing a single lesson. A significant negative correlation was found between anxiety and self-efficacy (r = −0.62, *p* < 0.01), a dynamic magnified under the spotlight of public lessons.

Professional Uncertainty: The polysemy of texts, the emergent nature of classroom interaction, and rapid technological iteration converge into a “triple cognitive load.” This manifests as 89% lacking confidence in interpreting culturally loaded terms, 34% torn between implementing task-based learning and teaching to the test, and 35% uncertain whether to address technical failures or salvage the lesson flow during smart classroom disruptions, fostering a state of chronic vigilance and fear of imminent loss of control.

### 3.8 Socio-cultural layers: cultural disciplining and projected expectations

Tension between Teacher Dignity and Dialogic Pedagogy: Confucian traditions demand that teachers maintain authority and model virtue (“be a paradigm of virtue and learning”), while the 2022 curriculum standards advocate “equal dialog” between teachers and students. The constant switching between “stooping to listen” and “standing to discipline” within a single lesson leads to “dual depletion of emotional labor.” Consequently, 28% resort to punitive point-deduction systems to maintain order, later regretting the sacrificed “emotional connection.”

Parental “Master-Teacher” Expectations and Performance-Oriented Evaluations: The pressure “not to betray parents’ trust” burdens 73% of novices. In 50.4% of the schools, performance in public lessons is a core annual appraisal metric, fostering “rehearsed performance teaching.” Furthermore, the linkage between monthly exam rankings and the district-controlled school appointment system compels teachers, despite recognizing the need for competency-oriented teaching, to revert to “knowledge-point cramming.”

In terms of the social media amplification effect, 35.89% of the teachers reported that “clipping errors in microlectures could lead to online bullying.” One case involved a teacher being forced into a “trending-topic apology” after a screenshot of their student’s writing feedback was taken out of context. This fostered compulsive self-censorship—"filtering every word three times mentally”—further eroding the safe space for spontaneous classroom interaction.

### 3.9 Institutional layers: disconnected training, alienated evaluation, and absent support

Echoing the findings of Du (2023) on evaluation anxiety, this study confirms the high-anxiety context of observed lessons, providing cross-disciplinary support for institutional safeguards such as “reducing the weighting of public lesson evaluations by 20%.”

Disconnected training system: Sixty-two percent of the respondents perceived a “complete disconnect” between university learning and classroom practice. Teacher education courses emphasize theories such as “New Criticism” and “Reception Aesthetics,” whereas frontline teaching requires “test-answering tactics” and “source-tracing of exam points.” Task-based unit design, covered in just two pages in textbooks, becomes a major responsibility upon employment, creating a methodological vacuum that directly fuels anxiety.

Instrumental Rationality in Evaluation: The consensus that “a public lesson is an examination hall” prevails, with leaders scoring onsite, peers photographing for records, and postlesson quantitative ranking. This leads teachers to dedicate 40% of their preparation time to “orchestrating applause” and “presetting eloquent soundbites.” The evaluation criteria overemphasize “lecture liveliness” and “lesson plan completeness” while neglecting “visibility of student thinking” and “competency appreciation,” which forces teachers into an “evaluation-instruction objective” conflict between fostering quality and chasing scores.

Structural Absence of Support Systems: Mentorship programs are effective for only 38% of teachers, and 33% of collective lesson preparation sessions devolve into “collective copying of lesson plans.” The usage rate of urban–rural shared resource platforms differs by 42 percentage points. Rural teachers, in addition to self-training in digital skills, often shoulder externally assigned administrative tasks, contributing to 24 percentage points higher technology anxiety than their urban counterparts do and creating a new digital divide characterized by “hardware in towns, software left behind.”

### 3.10 Disciplinary layer: intrinsic conflict between Chinese language characteristics and exam logic

It should be noted that the participants in this study are teachers of Chinese as a mother tongue, which shapes the nature of their anxiety in ways distinct from teaching Chinese as a foreign language.

Tension between Textual Openness and Standardized Answers: The inherent polysemy of classical Chinese, the suggestiveness of poetry, and the polyphony of novels are reduced to “single correct answers” on exams. This dilemma causes 89% of novices to be anxious about “covering too much (beyond the scope) or too little (superficial), “trapping them between “overinterpretation” and “amputating teaching for exams.”

Contradiction between task-based design and single-text depth: The new curriculum promotes “thematic task groups,” but weekly class hours remain unchanged. Consequently, 34.27% reported that “task groups feel like a patchwork,” squeezing out time for deep text analysis. Faced with the triple pressure of “unit integration, postlesson exercises, and secondary school entrance exam imperatives,” they vacillate between the “rhetoric of competency” and the “reality of drilling.”

Evaluation Vacuum for Cross-Media Writing: While new media forms such as “short video scripts” and “social media posts” enter textbooks, unified grading criteria are absent. Teachers fear that “creative writing” will not be recognized on exams and that “technical failures” will invite peer ridicule, ultimately retreating to the safe zone of “argumentative essay templates” and “memorized golden sentences,” thus intensifying the internal conflict between the subject’s “humanistic” and “instrumental” aims.

In summary, anxiety among novice Chinese language teachers results from the intricate intertwining of cognitive dissonance, cultural discipline, institutional neglect, and disciplinary tension. Isolated interventions are insufficient to dismantle this “volcanic structure.” A coordinated system addressing contextual buffering, instructional load reduction, technological integration, and institutional safeguards is essential to transform the heat of anxiety into energy for professional growth.

### 3.11 Theoretical contributions

Beyond delineating the specific causes of anxiety, this study makes two salient theoretical contributions that advance the field of teacher anxiety research.

First, we propose the ‘volcano model’ as a novel conceptual framework for understanding the structure and dynamics of teaching anxiety. As articulated in our findings, the volcano model conceptualizes teaching anxiety as comprising two interrelated components: the ‘magma chamber,’ representing latent psychological pressure accumulated through chronic stressors such as role conflict, perfectionism, and institutional demands; and the ‘eruption,’ referring to acute, situational anxiety episodes triggered by high-stakes events such as public demonstration lessons or leadership observations. These eruptions are not random but are activated when latent pressure exceeds a threshold in the presence of specific contextual triggers. This model shifts the focus from merely quantifying anxiety levels to understanding its underlying structure and activation mechanisms.

Second, the “identity-emotion-institution” tripartite framework offers a more integrated and powerful lens for analyzing teacher anxiety than previous, often siloed, approaches do. By conceptualizing anxiety as the product of a dynamic interplay between the cognitive-psychological (the teacher’s self-concept and efficacy), the socioemotional (the labor of managing one’s feelings according to cultural scripts), and the structural-institutional (the material conditions and evaluative logics of the workplace) perspectives, our framework moves beyond reductive explanations. The framework challenges the dichotomy of viewing anxiety as either an individual’s psychological weakness or a simple result of systemic pressure; instead, it demonstrates how these three layers are mutually constitutive: institutional demands trigger identity conflicts that require intensive emotional labor, the strain of which is in turn exacerbated by a lack of institutional support. This integrated model provides a comprehensive blueprint for future research, urging scholars to simultaneously consider these intertwined dimensions.

In conclusion, these theoretical innovations—the integrated tripartite framework and the dynamic volcano model—equip the field with more sophisticated conceptual tools. They not only deepen our understanding of anxiety among novice Chinese language teachers but also offer a transferable paradigm for investigating the complex emotional realities of teaching in other disciplinary and cultural contexts.

### 3.12 Implications for policy and teacher education

The empirically validated interventions and the support model proposed in this study translate directly into actionable recommendations for systemic reform in teacher education and educational policy.

First, our findings call for a foundational restructuring of teacher induction programs. The documented efficacy of the “teaching experience-adapted support” model, which reduced anxiety and increased self-efficacy, necessitated a shift away from one-size-fits-all induction. Policy-makers and school administrators should mandate phased support systems that align with the novice anxiety curve: a protected survival phase (0–6 months) with guaranteed workload limits, a structured development phase (6–18 months) with microcredentials for core skills, and a professionalization phase (18 + months) with fast-tracked career pathways. The success of the “microachievement system” (self-efficacy +39%) further suggests that teacher assessment during induction should move beyond high-stakes observations to include the validation of small, cumulative professional wins.

Second, the study provides compelling evidence for targeted investment and policy mandates. The dramatic reduction in anxiety and turnover intention following interventions such as “coteaching for inclusive classrooms” (interruptions −42%, turnover intent −28%) and “emotional leave” (turnover −28%) offers policy-makers a cost–benefit argument for funding specific support structures. We recommend the following educational authorities:

“Novice Teacher Well-being Grants” should be established to fund resource teachers, technology assistants, and mental health support in schools, with a particular focus on closing the rural–urban gap.

Institutionalize “emotional leave” policies at the district or provincial level, recognizing psychological well-being as a core component of professional sustainability.

Reform evaluation protocols by capping the weighting of public demonstration lessons in performance reviews, as our data conclusively show that reducing this weight by 20% can lower anxiety by 42%.

Finally, our results demand a reconciliation between preservice teacher education and in-service realities. The stark theory-practice gap reported by 62% of participants underscores systemic failure. Teacher training institutions must:

Integrate “high-context” pedagogical tools, such as the “Prism Interpretation Toolkit” (which reduced content anxiety by 58%), directly into their methodology courses.

Prioritize resilience and emotional competency training, simulating high-pressure scenarios such as public lessons and technology failures in a low-stakes environment to build psychological preparedness.

Forge stronger partnerships with schools to ensure that the cognitive calibration and contextual buffering strategies that proved effective for in-service teachers are introduced during preservice training.

In essence, this study provides a clear, data-driven roadmap. It argues that investing in nuanced, systemic support for novice teachers is not merely an act of compassion but also a strategic imperative for retaining talent, fostering instructional innovation, and ensuring the long-term health of the education system.

### 3.13 Dialog with international theoretical frameworks

The findings of this study resonate with yet also complicate several prominent international theoretical frameworks. First, our results strongly affirm the core tenets of the job demands-resources (JD-R) model. The intense anxiety triggered by evaluation and high teaching loads clearly represents salient job demands, while the success of our support model demonstrates the critical buffering role of job resources such as contextual buffering (e.g., mentorship, technical support) and institutional safeguards (e.g., emotional leave, workload protection). Our contribution extends the JD-R model by identifying and operationalizing a set of culturally and disciplinarily specific demands and resources. For example, the demand for “textual polysemy anxiety” and the resource of “expert mentorship within a collectivist culture” represent nuanced constructs that are paramount in the Chinese educational context but may be less salient in Western settings.

Second, our identified “teaching experience–anxiety curve” aligns with and challenges Fuller and Bown’s stage model of teacher concerns (Fuller and Bown, 1975). The initial peak in the first month corresponds neatly with the “self-survival” stage. However, the significant “second peak” of professional development anxiety at approximately 18 months reveals a more complex, nonlinear trajectory. This suggests that the transition from self-concerns to task and impact concerns is not a clean, sequential process. Instead, novice teachers appear to experience a layering of concerns, where survival anxieties are compounded, rather than replaced, by new pressures related to career advancement and sustainable growth. These findings corroborate and provide empirical support for Aldrup, K., Carstensen, B., & Klusmann observation of shifting emotional phases, suggesting that contemporary support systems must be dynamic and adaptable rather than assume a linear process of acclimatization (Aldrup et al., 2024).

Finally, by constructing the tripartite “identity-emotion-institution” framework, this study integrates psychological, sociological, and institutional perspectives on anxiety in a way that addresses calls in international scholarship for more integrated approaches to understanding teacher well-being (Zembylas, 2007). It moves beyond dichotomous debates of whether anxiety is an individual or systemic fault, instead demonstrating their synergistic interplay, a theoretical advancement with potential applicability beyond the Chinese context.

### 3.14 Limitations and future directions

This study has several limitations. First, the sample was drawn exclusively from Guangdong Province, which may limit the generalizability of findings to other regions. Second, while the mixed-methods design enhances validity, self-reported anxiety may still be subject to social desirability bias. Third, the intervention experiment, though rigorously designed, could not fully control for the natural accumulation of teaching experience over time, which may have contributed to reductions in anxiety. Future research should employ longitudinal designs with control groups across multiple regions and consider the use of physiological measures to complement self-report data.

## 4 Implications

Recognizing anxiety as both an individual signal and a systemic issue, this study proposes a comprehensive support framework based on the three identified characteristics-situational outbursts, teaching experience residuals, and individual heterogeneity. This framework comprises four interconnected components, each addressing a distinct mechanism of anxiety generation and alleviation:

Contextual buffering refers to interventions designed to reduce anxiety by modifying the environmental and situational conditions that trigger acute stress, such as public demonstration lessons or leadership observations.
Instructional load reduction involves structural adjustments to teaching hours, administrative duties, and preparation expectations to alleviate chronic workload pressure.
Technological integration encompasses the use of digital tools and platforms to support teaching, reduce uncertainty, and enhance instructional efficacy.
Institutional safeguards include formal policies and practices—such as emotional leave, reformed evaluation criteria, and mentorship systems—that protect novice teachers from excessive psychological and professional demands.

These four components are operationalized through a synergistic “cognitive-socioinstitutional” model, forming an actionable intervention loop grounded in systemic change.

### 4.1 Contextual buffering: transforming high-intensity triggers into manageable events

Cross-disciplinary evidence supports structured interventions for public lessons. For instance, studies on performance anxiety in teacher evaluations highlight the efficacy of simulation and feedback in reducing stress (Yada et al., 2022).

Lesson polishing clubs: collaborative preparation and scripted rehearsals focused on key transitions (introduction, bridging, evaluation) two weeks in advance reduced overpreparation time in experimental schools from 12.7 to 7.2 h per lesson.

Simulated studio with AI feedback: 4 K recording combined with AI analysis of 22 behavioral indicators (e.g., pauses >2 s, filler words, gaze aversion) provided real-time feedback. Teachers accessed personalized “anxiety heatmaps,” leading to a 31% increase in high-quality lesson delivery.

Expert mentorship: teaching-researchers provided nonevaluative, constructive feedback within 30 min post-lesson, focusing solely on improvement. This approach reduced associated anxiety scores by 37%.

Technical contingency plans (“Red Protocols”): provincially standardized “10-s restore” microlesson packages were developed for common failures (internet outages, black screens, and audio loss). Rural schools were prioritized for “Technical Assistant” support, which reduced technological anxiety during public lessons from 4.8 to 2.1.

Coteaching for classrooms with special needs: for classes with >15% of students having learning or behavioral difficulties, county funding supported the addition of resource teachers (1:0.3 teacher-class ratio) or AI-based early warning services. This reduced classroom interruptions by 42% and teacher turnover intention by 28%.

### 4.2 Teaching experience-adapted support: dynamic alignment with the anxiety curve

#### 4.2.1 Survival phase (0–6 months)

Workload protection: a maximum of 10 weekly teaching periods and ≤30% homeroom advisor roles for the first three years; administrative form completion was performed centrally.

Microachievement system: teaching goals were broken down into 20 quantifiable microtasks (e.g., successfully leading a group discussion). Instant visual feedback increased self-efficacy by 39%.

#### 4.2.2 Development phase (6–18 months)

Staged growth ladder (“1–3-5”): a structured focus on classroom survival (Week 1), core skills (Months 1–3), and task-based design (Months 4–5).

Prism interpretation toolkit: providing historical annotations, curriculum standard analyses, and classroom examples for the same text reduced text interpretation anxiety by 58%.

#### 4.2.3 Professionalization phase (18 + months)

Fast-track professional promotion: dedicated quotas (20%) for novices, accepting microlessons, innovation cases, and teaching narratives as alternatives to academic papers.

AI-powered anxiety alert: integrating 22 data points (voice, expression, heart rate) to trigger automated mindfulness audio and demonstration videos when anxiety levels exceed a threshold (STAI>6), increasing intervention success by 56%.

### 4.3 Technological integration: bridging the urban–rural divide

Provincially managed co-teaching pools: a total of 1,000 urban master teachers were selected annually for coteaching partnerships with rural schools, combining live streaming and site visits. This increased rural students’ access to high-quality instruction from 1.2 to 3.5 lessons/week and narrowed the gap in rural–urban technology anxiety to 8%.

Technical assistant redeployment plan: art and PE teachers received two weeks of training in equipment maintenance and resource editing. Certified “Technical Assistants” managed school equipment and supported online Q&As, reducing county-wide equipment failure rates by 63%.

Integrated cloud-based teaching-research platform: a platform hosting 200,000 + lesson examples, 5,000 + microlessons, and AI diagnostic tools used a “tag-push-peer review” algorithm. Rural teachers’ resource access efficiency increased fivefold, and pro-forma collective lesson preparation decreased from 33 to 11%.

### 4.4 Institutional safeguards: novice protection and evaluation reform

Quantifying the risk of high teaching loads (OR = 1.97 for ≥13 weekly lessons) and demonstrating that reducing the evaluation weight of public lessons by 20% lowers anxiety by 42% provides precise evidence for institutional reform.

Emotional Leave and psychological safety: in pilot schools, the implementation of two paid ‘emotional leave’ days per month—available for self-directed activities such as counseling, peer observation, or well-being workshops—resulted in a 27% reduction in burnout and a 28% decrease in turnover intention, demonstrating the protective effect of institutionalized psychological support.

Restructured evaluation metrics: moving beyond macrolevel proposals (Xu et al., 2025), this study pilots specific metrics such as “student thinking visibility” and “competency,” implementing a “Three-Dimensional Value-Added Assessment.” This reduced teacher anxiety by 42% and increased teaching innovation by 31%.

Provincial funding coordination: a dedicated “Novice Teacher Anxiety Intervention Fund” was established, with provincial-level financing for economically disadvantaged counties to ensure sustainable implementation.

### 4.5 The synergistic “cognitive–socioinstitutional” model

#### 4.5.1 Cognitive layer: calibration and empowerment

Dual-loop empowerment: “Theory-practice” discussions coupled with microachievement accumulation reduced self-assessment bias by 41%.

Knowledge support matrix: parallel databases of “historical annotations, curriculum standards, teaching cases” increased task-based unit completion rates by 63%.

#### 4.5.2 Socio-cultural layer: expectation management and cultural reshaping

Role-playing workshops: simulating “authoritative, dialogic, hybrid” classroom cultures improved teachers’ cultural adaptability by 58%.

Parent education initiative: parent school courses address “master-teacher worship,” increasing satisfaction rates from 61 to 89%.

#### 4.5.3 Institutional layer: vertical integration and process oversight

Decentralized management: delegating educational supervision to township governments.

Centralized financial security: establishing a four-tier (province-city-county-township) cost-sharing mechanism.

Third-party process evaluation: annual audits of intervention projects by universities and professional associations linked to government performance evaluations.

The implementation of this integrated framework in experimental schools yielded significant results: the average STAI score decreased from 5.1 to 2.4, turnover intention decreased by 28%, and the number of teaching innovation cases tripled. This provides an evidence-based, data-driven paradigm for constructing a targeted teacher support system.

### 4.6 Theoretical implications

The findings of this study not only provide a rich, situated understanding of anxiety among novice Chinese language teachers but also carry significant theoretical implications, engaging in a constructive dialog with established models in the fields of occupational stress and teacher development.

First, this study validates and extends the seminal job demands-resources (JD-R) model (Bakker and Demerouti, 2007). Our findings powerfully illustrate the “job demands” specific to this population: intense evaluation and assessment anxiety and instructional load represent salient psychological demands, whereas student behavioral issues and technological challenges constitute key organizational and work-environment demands. Conversely, the support model we developed and validated, encompassing contextual buffering, institutional safeguards, and technological integration, operates as a critical bundle of “job resources.” The demonstrated reduction in anxiety and turnover intention when these resources are provided offers robust empirical support for the core JD-R premise that resources can buffer the negative impact of demands. Our contribution lies in extending this model by identifying and meticulously detailing the unique, discipline-specific demands (e.g., textual polysemy, the “overinterpretation vs. exam” dilemma) and culturally situated resources (e.g., “expert,” emotional leave within a Confucian context) that are paramount in the Chinese educational setting.

Second, our findings on the nonlinear “teaching experience-anxiety curve” both resonate with and complicate traditional stage models of teacher development, such as Fuller and Bown's (1975) model of teacher concerns. The initial survival anxiety peak aligns with their “self-concerns” stage, where teachers are preoccupied with their own performance and adequacy. However, the emergence of a “second peak” of professional development anxiety at approximately 18 months reveals a more complex trajectory; it suggests that the transition from “self-concerns” to “task concerns” and “impact concerns” is not a simple, linear progression. Instead, novice teachers may experience a layering of concerns, where survival anxieties do not fully dissipate but are compounded by new pressures related to professional growth and career advancement. This “residual resurgence” challenges the linearity of stage models and underscores the need for a more dynamic, nonlinear conceptualization of the novice teacher’s emotional journey.

Finally, the construction of our “identity-emotion-institution” explanatory framework and the “volcano model” of the anxiety structure represents a theoretical innovation. It moves beyond one-dimensional psychological or sociological explanations to propose a multilayered, interactive theoretical framework. This framework challenges reductionist accounts of teacher anxiety by demonstrating how cognitive (the ideal self), sociocultural (emotional labor norms), and institutional (evaluation systems) forces are not merely additive but interact synergistically to produce the “multilayered fault” of anxiety. By integrating the disciplinary layer as a core axis, our model further asserts that the experience of teaching anxiety cannot be fully understood without considering the unique epistemological and pedagogical characteristics of the subject being taught.

In summary, this study does more than just document a problem; it engages with and advances theory. It validates the JD-R model in a novel context, refines developmental stage theory with empirical nuance, and proposes a new, integrated framework for understanding the complex, situated nature of anxiety that may inform future research beyond the field of language teacher education.

## 5 Conclusion

This mixed-methods study moves beyond speculative intervention proposals (Yang, 2021) by constructing and empirically validating a replicable support framework for novice Chinese language teachers’ anxiety. Grounded in robust evidence from 1,248 questionnaires, 260 open-ended responses, our findings confirm that teaching anxiety is not a monolithic construct but a dynamic, situational phenomenon. It is characterized by a “low-baseline, high-outburst” volcanic structure, primarily triggered by public demonstrations, leadership observations, and the management of students with special needs. The nonlinear “teaching experience–anxiety curve,” marked by a sharp rise, a gradual decline, and a significant residual resurgence at approximately 18 months, highlights a paradigm shift from static to dynamically adaptive support systems.

Theoretically, this study makes two salient contributions with respect to international resonance. First, the tripartite “identity-emotion-institution” explanatory framework challenges reductionist accounts of anxiety as either a personal failing or a mere systemic byproduct. By demonstrating how cognitive ruptures in the ideal self, sociocultural disciplining of emotional labor, and institutional voids interact synergistically, we provide an integrated lens through which teacher anxiety in other high-stakes, culturally specific contexts can be examined. Second, the “volcano model” offers a powerful metaphor and analytical tool for understanding the complex structure of anxiety itself. These findings underscore that a low level of resting anxiety can be dangerously deceptive, as immense pressure builds beneath the surface and erupts in specific high-stakes situations. This model shifts the research focus from merely quantifying anxiety levels to deciphering its underlying triggers, temporal dynamics, and moderating risk factors, thereby informing the design of more precise and preemptive interventions.

In terms of policy and practice, the empirically validated success of our four-dimensional intervention model—Contextual Buffering, Instructional Load Reduction, Technological Integration, and Institutional Safeguards—provides a clear, actionable roadmap. The dramatic reductions in anxiety (STAI scores from 5.1 to 2.4) and turnover intention (−28%) achieved through measures such as the three-tier public lesson support system, emotional leave, and the urban–rural coteaching pool offer compelling evidence for systemic investment. Policy-makers and educational administrators must act on these findings as follows:

Mandating phased induction programs aligned with the anxiety curve, including workload protection in the survival phase and fast-tracked professional pathways after 18 months.

Institutionalizing psychological safeguards, such as emotional leave and access to mental health resources, as nonnegotiable components of teacher well-being.

Reforming evaluation systems to reduce the high-stakes weight of public lessons and incorporate metrics that value student thinking and competency development.

A primary limitation of this study is its geographic focus on Guangdong Province. Future research should employ multiregional sampling to test the generalizability of the “volcano model” and the proposed framework. The complex, evolving nature of teacher anxiety also calls for innovative research methodologies. We propose the development of a “digital twin system for teacher anxiety,” leveraging real-time, multimodal data (e.g., voice, biometrics) to enable personalized, just-in-time intervention. Ultimately, transforming the latent “crater” of anxiety into a sustainable “hot spring” of professional growth requires a synergistic ecosystem that includes data-informed governance, institutional courage, and a culture of collective support. Only through such a concerted effort can we guide novice Chinese language teachers—and potentially their counterparts globally—from a state of being “constantly on the verge of eruption” to one of sustained, fulfilling professional practice.

## Data Availability

The raw data supporting the conclusions of this article will be made available by the authors, without undue reservation.
