# Supplementary material for: Teaching anxiety among novice Chinese language teachers: manifestations, causes, and interventions—a mixed-methods study in Guangdong Province, China
Source: Front Psychol. 2026 Mar 25;17:1796834. doi: 10.3389/fpsyg.2026.1796834 (PMC13057351; doi:10.3389/fpsyg.2026.1796834)
Supplement: Supplementary file 1 [file Supplementary_file_1.pdf]

Appendix A

Survey on Teaching Anxiety Among Novice Chinese Language Teachers (Integrated Version)

Part One: Basic Information

1. Your teaching experience:

☐0－3 months ☐4－6 months ☐7－12 months ☐1－2 years ☐3+ years

2. Teaching Level:

☐ Primary School ☐ Junior High School ☐ Senior High School ☐ Cross-Level Teaching

3. Educational Background:

☐ Chinese Language and Literature ☐ Bachelor’s Degree ☐ Master’s Degree ☐ Doctoral Degree ☐ Other Major

4. Teacher training student: ☐ Yes ☐ No

5.Intern or not:☐ Yes ☐ No

6. School Type: ☐ Public ☐ Private ☐ Other

7.The school is located at: ☐City ☐County town ☐Township☐Rural

8. Average weekly class hours:

☐Less than 10 classes ☐11－15 classes ☐16－20 classes ☐20 or more classes

9. WhetherAt the same timeClass advisor: ☐ Yes ☐ No

Part Two: Manifestations of Teaching Anxiety

A. Pre-class anxiety group

10. A text lessonPreparation time: ☐ ≤ 1 hour ☐ 1－2 hours ☐ 2－3 hours ☐ ≥ 4 hours

11. Lesson Preparation Focus (Multiple Choice):

☐ Accuracy of instructional content ☐ Students’ cognitive level ☐ Design of classroom interaction ☐ Integration of multimedia resources ☐ Contingency plans for unexpected situations

12.Depth of interpretation of the textbook text during lesson preparation:

☐ Completely reliant on teaching reference materials ☐ Able to provide personalized interpretations ☐ Often stuck in the dilemma of multiple interpretations ☐ Faces a clear risk of misinterpretation

13. Trial teaching frequency:

☐ Mandatory for every class ☐ Trial teaching for key classes ☐ Occasional trial teaching ☐ Never trial teach

14. Pre-class physiological responses (multiple choices):

☐ Sweaty palms ☐ Trembling voice ☐ Frequent urination ☐ Insomnia ☐ Loss of appetite

B. Classroom Anxiety Group

15. Classroom Tension Index (1-10 points),The higher the score, the more tense you are.）:

☐1-3 ☐4-6 ☐7-9 ☐10

16. Typical anxiety scenarios (multiple choices):

☐ Open class ☐ Observation by leaders ☐ Introduction to new lesson ☐ Question-and-answer session ☐ Handling disciplinary violations ☐ Multimedia malfunction

17. Knowledge Gap Response (Multiple Choice):

☐ Verify immediately and provide additional explanation ☐ Blur the details ☐ Change the subject ☐ Admit ignorance and offer a follow-up lecture later

18. Classroom Silence Response (Multiple Choice):

☐ Repeated question ☐ Call on students to answer ☐ Group discussion ☐ Self-questioning and self-answering ☐ Skip the question

C. Reflection Anxiety Group

19. Feedback processing after the observed class (single-choice):

☐ Record improvements item by item. ☐ Selective listening ☐ Selective ignoring ☐ Eliciting defensive reactions

20. Students' sensitivity to classroom evaluations (single-choice):

☐ Highly sensitive ☐ Relatively concerned ☐ Moderately concerned ☐ Not very concerned ☐ Not concerned at all

21. Post-class review duration:

☐None☐ ≤ 30 minutes ☐ 30－60 minutes ☐ 1－2 hours ☐ > 2 hours

D. Specialized Anxiety in Language Arts Teaching

22-1. The greatest challenge in teaching classical Chinese:

☐ Distinction between content words and function words ☐ Accuracy of sentence structure translation ☐ Integration of cultural and historical context ☐ Engagement of student interest

22-2. Modern Chinese Language Teaching Anxiety Scenarios:

☐ Inaccurate grasp of essay emotions ☐ Diversified interpretations of novel themes ☐ Difficulty in analyzing poetic imagery ☐ Boring teaching of practical texts

22-3. Pain points in writing instruction:

☐ The questions lack originality. ☐ Grading efficiency is low. ☐ Feedback lacks specificity. ☐ Students engage in plagiarism.

23. Difficulties in oral communication lessons:

☐ Insufficient student willingness to express themselves ☐ Vague evaluation criteria ☐ Interference from dialects ☐ Difficulty in guiding spontaneous conversations

24. The challenge of interdisciplinary integration:

☐ Task-group instructional design ☐ Guidance on reading entire books ☐ Implementation of cross-media reading ☐ Conducting project-based learning

25. Cultural Heritage Anxiety:

☐ Interpretation of Traditional Festival Culture ☐ Teaching Guidelines for Red Classics ☐ Value Guidance through Foreign Literary Works ☐ Impact of Internet Slang

26.Classroom discussion getting out of control:

☐ Deviation from the preset theme ☐ Student’s viewpoint is incorrect and needs correction ☐ Deep thinking sparks controversy ☐ Awkward silence

27.Weaknesses in my Chinese language skills:

☐ Recitation and dictation of classical poetry and prose ☐ Systematic knowledge of grammar and rhetoric ☐ Literary theory proficiency ☐ Ability to write in various literary styles

E. Anxiety about instructional evaluation

28. Pressure of homework grading:

☐ Time-consuming essay grading ☐ Subjective question scoring criteria ☐ Insufficient personalized feedback ☐ Difficulty in detecting plagiarism

29. Exam Question-Setting Anxiety:

☐ Risk of innovative question types ☐ Comprehensive coverage of exam points ☐ Control over discrimination power ☐ Pressure from performance rankings

30. Sensitive points in lecture feedback:

☐ Standardization of blackboard writing ☐ Vividness of classroom language ☐ Completeness of teaching activities ☐ Proficiency in using multimedia

F. Student Management Anxiety

31. Challenges of Student Learning Differences:

☐ Language proficiency differences between urban and rural students ☐ Teaching Mandarin in dialect regions ☐ Transforming underperforming students ☐ Bottlenecks in enhancing the abilities of top-performing students

32. Behavioral Management Conflict:

☐ Classroom chat disrupting teaching ☐ Mobile phone usage restrictions ☐ Confrontational teacher-student interactions ☐ Communication with students from special family backgrounds

33. Time spent on discipline maintenance:

☐ > 20% of class time ☐ 10-20% of class time ☐ < 10% of class time ☐ No significant impact

34. Proportion of students with special needs: ☐ ⩾30% ☐ 15-30% ☐ <15% ☐ No students with special needs

35. Frequency of home-school communication: ☐ Daily ☐ Weekly ☐ Monthly ☐ At the beginning/end of the semester ☐ Rarely

G. Professional Development Anxiety

36. Self-improvement dilemma:

☐ Disconnection between educational theory and practice ☐ Writing research papers ☐ Ability to apply for research projects ☐ Pressure of demonstrating public lessons

37. Digital Teaching Anxiety:

☐ Smart Classroom Technology Operation ☐ Micro-lesson Production Skills ☐ Adaptability to AI-Assisted Teaching ☐ Use of Electronic Grading Systems

38. Textbook familiarization period: ☐ ⩽ 1 month ☐ 2－3 months ☐ 4－6 months ☐ ⩾ 1 academic year

39. Dependence on supplementary teaching materials: ☐ Completely dependent ☐ Relatively dependent ☐ Moderately referenced ☐ Independently designed

40. Information Technology Application Skills: ☐ Proficient ☐ Basically proficient ☐ Initially familiar ☐ Not yet proficient

41. Pressure for promotion: ☐ Extremely high ☐ Relatively high ☐ Moderate ☐ Relatively low ☐ None

42. Workload of teaching and research tasks: ☐ Overloaded ☐ Relatively heavy ☐ Moderate ☐ Relatively light ☐ None

Part Three: Diagnosis of Anxiety Triggers

A. Career Development Dimension

43. Effectiveness of the mentorship program:

☐ Very helpful ☐ Somewhat helpful ☐ Limited help ☐ Virtually useless

B. Organizational Environment Dimension

44. Principal’s level of support:

☐ Full support ☐ Relatively supportive ☐ Average ☐ Less supportive ☐ No support

45. Peer Competition Intensity:

☐ Intense ☐ Relatively Strong ☐ Moderate ☐ Relatively Weak ☐ No Competition

46. Rationality of the evaluation system:

☐ Very reasonable ☐ Relatively reasonable ☐ Average ☐ Relatively unreasonable ☐ Extremely unreasonable

C. Triggering Events and Persistence

47. The primary anxiety-triggering event:

☐ Open class teaching ☐ Teaching incident ☐ Parent complaint ☐ Performance evaluation and recognition ☐ Peer competition

48. Characteristics of anxiety duration:

☐ Intense outbreak before exams ☐ Start of the new semester ☐ Prolonged chronic stress ☐ Triggered by occasional events

Part Four: Demand for Mitigation Strategies

A. Individual regulatory strategies

49. Meditation’s stress-reducing effects:

☐ Significant ☐ Effective ☐ Moderate ☐ Ineffective ☐ Not tried

50. Exercise frequency:

☐ Daily ☐ 3-4 times per week ☐ 1-2 times per week ☐ Occasionally ☐ Never

51. Reading Therapy Preferences (Multiple Choice):

☐ Educational Psychology ☐ Collection of Famous Teachers' Cases ☐ Poetry and Prose ☐ Professional Journals ☐ Leisure Reading

52. Ranking of the effectiveness of anxiety relief methods:

☐ Self-reflection ☐ Peer discussion ☐ Expert guidance ☐ Physical exercise for emotional release ☐ Psychological counseling

B. Organizational Support Strategies

53. Mentorship Program Improvement Needs:

☐ Urgently Needed ☐ Needed ☐ Moderate ☐ Not Needed ☐ Completely Unnecessary

54. Effectiveness of Collaborative Lesson Planning:

☐ Highly Effective ☐ Relatively Effective ☐ Formalistic ☐ Ineffective ☐ No Effect

55. Availability of psychological counseling services:

☐ Convenient ☐ Average ☐ Inconvenient ☐ Absent ☐ Unknown

C. Institutional Optimization Strategies

56. Demand for flexible class scheduling:

☐ Urgent ☐ Needed ☐ Moderate ☐ Not needed ☐ Opposed

57. New Teacher Workload Reduction Policy:

☐ Very Necessary ☐ Necessary ☐ Optional ☐ Not Necessary ☐ Opposed

58. Fairness of resource allocation:

☐ Highly recognized ☐ Somewhat recognized ☐ Average ☐ Less recognized ☐ Not at all recognized

Part 5: Open-ended Questions

59. Please describe the most memorable instance of classroom anxiety you've experienced and the process you went through to deal with it.

____

____

60. Please list three teaching scenarios that cause you the most anxiety:

____

61. What do you think is the most effective support measure for addressing language arts teachers' anxiety?

____

62. The targeted support measure you’ re most looking forward to is:

____

63. The greatest expectation for Chinese language education policies or training systems:

____

64. What do you think is the area that school management should most improve?

____

I have successfully merged the two questionnaires into one that is logically organized and comprehensively detailed. The integrated questionnaire contains a total of 64 questions, grouped by module as follows:

Basic Information(7 questions): Covers basic information including years of teaching experience, teaching level, educational background, whether the individual is a teacher trainee, type of school, average weekly class hours, and whether the individual serves as a homeroom teacher.

Manifestations of Teaching Anxiety(Question 27):

1.Pre-class anxiety group: preparation duration, key areas of focus during preparation, frequency of practice teaching sessions, pre-class physiological responses, and depth of text interpretation.

2.Classroom Anxiety Group: Classroom Stress Index, Typical Anxiety Scenarios, Addressing Knowledge Gaps, Dealing with Classroom Silence

3.Reflection Anxiety Group: Processing of Class Feedback, Student Evaluation Sensitivity, Duration of Post-Class Debriefing

4.Specialized Study on Language and Literature Teaching Anxiety: Challenges in Classical Chinese Instruction, Anxiety in Modern Chinese Instruction, Loss of Control in Classroom Discussions, Pain Points in Writing Instruction, Difficulties in Oral Communication, Weaknesses in Foundational Language Skills, Anxiety About Cultural Heritage, and Challenges in Interdisciplinary Integration.

5.Teaching Evaluation Anxiety: Pressure from Grading Assignments, Anxiety About Exam Question Design, and Sensitivity to Feedback During Class Observations

6.Student Management Anxiety: Challenges Posed by Learning Differences, Conflicts in Behavior Management, Time-Consuming Discipline Maintenance, Proportion of Students with Special Needs, and Frequency of Home-School Communication

7.Professional Development Anxiety: Challenges in Self-Improvement, Anxiety About Digital Teaching, Duration of Familiarization with Textbooks, Dependence on Supplementary Teaching Materials, Competence in Applying Information Technology, Pressure to Advance in Professional Titles, and Burden of Research and Teaching Tasks

Anxiety Trigger Diagnosis(8 questions):

1.Career Development Dimension: Effectiveness of the Mentorship System

2.Organizational Environmental Dimensions: Principal Support, Peer Competition Intensity, and Rationality of the Evaluation System

3.Triggering Events and Duration: Major Anxiety Triggering Events and Characteristics of Anxiety Duration

Relief Strategy Needs(8 questions):

1.Individual Regulation Strategies: Ranking of the Effectiveness of Meditation for Stress Reduction, Frequency of Exercise-Based Regulation, Preferred Healing Through Reading, and Methods for Anxiety Relief

2.Organizational Support Strategies: Mentorship Program for Enhanced Needs, Effective Collective Lesson Preparation, and Accessibility of Psychological Counseling Services

3.Institutional Optimization Strategies: Demand for Flexible Class Scheduling, Policies to Reduce Workloads for New Teachers, and Fairness in Resource Allocation

Open-ended question(6 questions): Includes descriptions of classroom anxiety incidents, lists of anxiety-inducing scenarios, and suggestions for supportive measures.

This integrated questionnaire fully leverages the strengths of the two original questionnaires, covering both teaching anxiety specific to language arts and general manifestations, causes, and coping strategies for teaching anxiety, thereby forming a comprehensive survey system.
